# Decoding lip language using triboelectric sensors with deep learning

**DOI:** 10.1038/s41467-022-29083-0

**Published:** 2022-03-17

**Authors:** Yijia Lu, Han Tian, Jia Cheng, Fei Zhu, Bin Liu, Shanshan Wei, Linhong Ji, Zhong Lin Wang

**Affiliations:** 1 State Key Laboratory of Tribology, Department of Mechanical Engineering, Tsinghua University, Beijing, 100084 China; 2 National Laboratory of Pattern Recognition, Institute of Automation, Chinese Academy of Sciences, Beijing, 100190 China; 3 Beijing Institute of Nanoenergy and Nanosystems, Chinese Academy of Sciences, Beijing, 101400 China; 4 School of Nanoscience and Technology, University of Chinese Academy of Sciences, Beijing, 100049 China; 5 School of Materials Science and Engineering, Georgia Institute of Technology, Atlanta, GA 30332-0245 USA

**Keywords:** Materials for devices, Electronics, photonics and device physics

## Abstract

Lip language is an effective method of voice-off communication in daily life for people with vocal cord lesions and laryngeal and lingual injuries without occupying the hands. Collection and interpretation of lip language is challenging. Here, we propose the concept of a novel lip-language decoding system with self-powered, low-cost, contact and flexible triboelectric sensors and a well-trained dilated recurrent neural network model based on prototype learning. The structural principle and electrical properties of the flexible sensors are measured and analysed. Lip motions for selected vowels, words, phrases, silent speech and voice speech are collected and compared. The prototype learning model reaches a test accuracy of 94.5% in training 20 classes with 100 samples each. The applications, such as identity recognition to unlock a gate, directional control of a toy car and lip-motion to speech conversion, work well and demonstrate great feasibility and potential. Our work presents a promising way to help people lacking a voice live a convenient life with barrier-free communication and boost their happiness, enriches the diversity of lip-language translation systems and will have potential value in many applications.

## Abstract (web summary)

Lip-language decoding systems are a promising technology to help people lacking a voice live a convenient life with barrier-free communication. Here, authors propose a concept of such system integrating self-powered triboelectric sensors and a well-trained dilated RNN model based on prototype learning.

## Introduction

Humans can use lip language for communication without audible acoustic signals generated from the speaker. Lip language can be used in the following typical scenarios: persons who have undergone laryngectomy only mouthing words rather than pronouncing them; covert conversations occurring on public occasions; and the speech process with interference in a high background noise environment. However, lip language is notoriously difficult for audiences to master, although it is user friendly for speakers. Most people can only understand a few words by lip reading. Decoding lip language easily and directly for audiences is a valuable challenge. Sign language^1^ is another widely used method for inaudible communications via vision, especially for hearing-impaired persons. Many studies have been carried out into sign language^2–5^. However, lip language has some advantages over sign language, such as lip language frees one’s hands in speech compared with sign language; lip language uses the existing human speech process while mastering sign language requires one to learn a new norm from the beginning; and lip language has more semantic diversity than sign language.

Researchers have performed many excellent studies into lip-language recognition^6,7^. Silent speech interfaces (SSIs)^8,9^ are systems enabling speech in inaudible scenarios, including magnet-based solutions^10–12^, vision-based solutions^13,14^, ultrasound-based solution^15^, inaudible acoustic-based solution^16^ and surface electromyography (sEMG) based solutions^17,18^. With the enhancement of rapidly developed machine learning and deep learning in recent years^19–23^, many works have achieved higher recognition rates than traditional algorithms. However, noncontact visual methods suffer interference from facial angles, light intensity, head shaking and blocking objects. When a speaker’s mouth is covered by an opaque mask, as is the case in an epidemic of respiratory infectious diseases (currently, the coronavirus disease 2019 (COVID-19) pandemic), vision-based solutions fail. Lip language is demonstrated by a series of mouth shapes^13,14^, also known as the motion of essential mouth muscles. Non-invasive and contact sensors capturing the motion of muscles are immune to the problems of the vision-based solutions encountered above, which is a promising way of acquiring data from muscle movement.

Recently, triboelectric nanogenerators (TENGs)^24–26^ have attracted increasing attention worldwide. TENGs based on charge electrification (CE) and electrostatic induction convert tiny mechanical energy into electricity and harvest scattered mechanical energy^27,28^. TENG-based sensors^29–31^ are low-cost, self-powered and generate electrical signals without an additional power supply source. Self-powered characteristics help simplify the circuit structure for sensor data acquisition. Studies of TENG-based sensors in human motion detection^32–34^, human-computer interaction^35–40^, respiratory^41^, vibration^42^ and sound^43,44^ detection, tactile^45,46^ and pressure sensing^47,48^ and wearable detection devices have been openly reported.

In this article, we propose a novel lip-language decoding system (LLDS) for capturing motions of mouth muscles aided by flexible, low-cost and self-powered sensors and recognizing signals with a deep learning classifier. Self-powered sensors placed into the junction of mouth muscles are fabricated with flexible polymer films to improve the sensation of the skin on the mouth. A dilated recurrent neural network model based on prototype learning is adopted to cope with the challenges of signal diversity and personalized small samples and test accuracy of 94.5% is reached. A mask is chosen to assist the positioning and fixation of the sensors, which increases privacy and confidentiality and interrupts the transmission of respiratory infectious diseases. This approach opens up a new possibility for translating lip motion to speech or text directly and conveniently by capturing muscle movements providing assistance to people with a missing voice with few resources occupied. This work presents a promising way to help people with speech disorders live a happy life with barrier-free communication and will enrich the diversity of lip-language translation systems in human-machine interfaces and silent speech interfaces.

This article is arranged as follows: the structure and components of the LLDS and its workflow, structure and mechanism of the triboelectric sensors attached around the mouth are described in detail. The fundamental characteristics of the triboelectric sensors, such as open-circuit voltage, short-circuit current, series-parallel connection, load curve and durability, at different forces and frequencies are carefully investigated. The lip-motion signals for typical words are collected and compared with the synchronized sound. The impact of speaking speed and lip-motion pattern on the lip signals, signals of silent speech and voiced speech are compared and analysed. The time required for mouth motion ahead of the sounds heard is counted at specific words. The lip signals are sampled in a specific word group for training with a machine-learning (ML) model. The applications supported by LLDS, such as identity recognition to unlock a gate and directional control of the movement of a toy car, work well and demonstrate the potential feasibility of improving communication for people with speech barriers with lip language aided by triboelectric sensors.

## Results

### Design of the lip-language decoding system and the triboelectric sensors

**Fig. 1:**
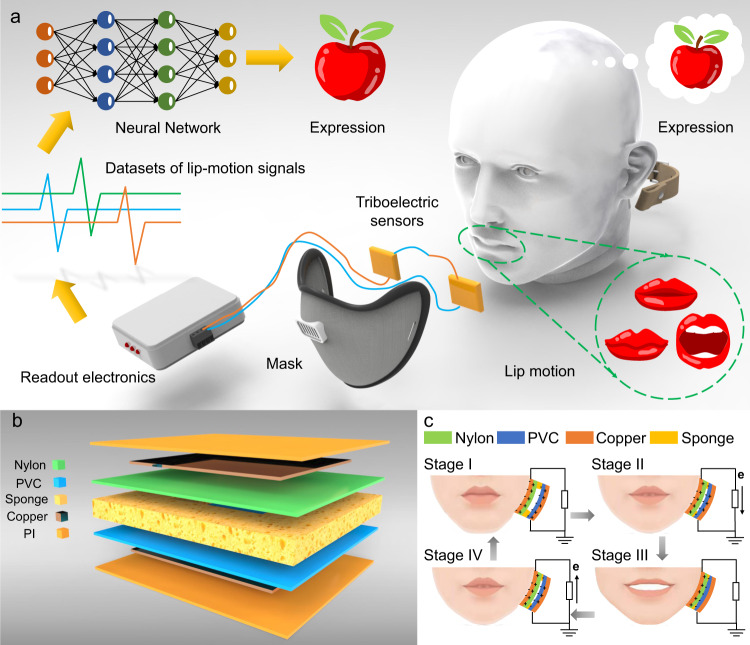
The concept, structure and mechanism of the lip-language decoding system supported by triboelectric sensors. **a** Schematic illustration of the lip-language decoding system and its components, including triboelectric sensors, signal processing and deep learning classifiers. **b** Structure scheme for the flexible triboelectric sensor. **c** Schematic diagram of four stages of charge transfer in one mouth open-close cycle. The mouth-opening process pushes the sensor, and the mouth-closing process releases the sensor, resulting in the flow of current in opposite directions.

Here, we propose the concept of the lip-language decoding system (LLDS) supported by flexible triboelectric sensors to capture signals and use a deep learning-assisted classifier to translate the lip language, as shown in Fig. 1a. The system is composed of triboelectric sensors, fixing masks, readout electronics, and neural network classifiers. Flexible triboelectric sensors are placed at the junction of the orbicularis oris, depressor anguli oris, risorius, zygomaticus and buccinator to capture muscle motions. One can take the process of a person saying the word ‘apple’ as an example. When he or she speaks, the triboelectric sensor detects the movements of the lip muscles and generates a series of electrical signals. The relevant electronic equipment reads out the electrical signals and performs routine processing. Finally, the signals due to the lip muscle movements are recognized following transfer to the trained neural network, and the recognized information is transmitted by sound or text on a screen. A mask is used to assist with locating and fixing the triboelectric sensors at specific positions corresponding to the relevant mouth muscles. Both the mask and sponge help to provide pretension to adjust the amplitude of the signals.

The structure scheme for the triboelectric sensor is shown in Fig. 1b. This is a typical contact-separation mode for TENGs with double electrode structures. The sponge in the middle of the sensor recovers its shape when the compression disappears. A rectangular hole cut in the centre of the sponge is used to complete the charge transfer via contact of the materials. Polyvinyl chloride (PVC) and polyamide (nylon) films are distributed on both sides of the sponge and covered by copper films. The sensor is packaged with polyimide (PI) film to keep human skin, sweat, and skin flakes away from the copper electrodes. The materials used to build the sensor are low-cost and easily obtained. The components of the sensor are thin plastic films, sponge and thin copper foils, which are flexible to fit the deformation of facial muscles.

The working mechanism of electric signal generation for the triboelectric sensor is shown in Fig. 1c. Electric charges are generated by contact and separate motion of materials; PVC gains electrons, and nylon loses electrons. The charge transfer in one mouth open-close cycle (pressing and releasing) process is divided into four stages. No charge changes during stage I when the mouth remains closed, and nylon and PVC films induce corresponding positive and negative charges on the copper electrodes, respectively. When the mouth opens, the muscles press the sensor in stage II, and the nylon films move closer to the PVC. The induced charges on the relevant copper electrodes decrease, and the current flows from the PVC-side electrode to the nylon-side electrode. When the mouth opening reaches its maximum at stage III, the current is reduced to zero, and the charges induced on the electrodes decrease to almost zero. In mouth-closing stage IV, when the nylon film moves away from the PVC, the charges induced on the copper electrode increase, and the current flows from the nylon-side electrode to the PVC-side electrode. The sponge shifts to its original shape and enters stage I again, completing an entire mouth open-close cycle. The distributions of the electric field for the sensor at different press-releasing states are simulated by COMSOL, as shown in Supplementary Fig. 1, and from left to right correspond to stages I, II(IV), and III in Fig. 1c, respectively.

### Fundamental characteristics of triboelectric sensors

**Fig. 2:**
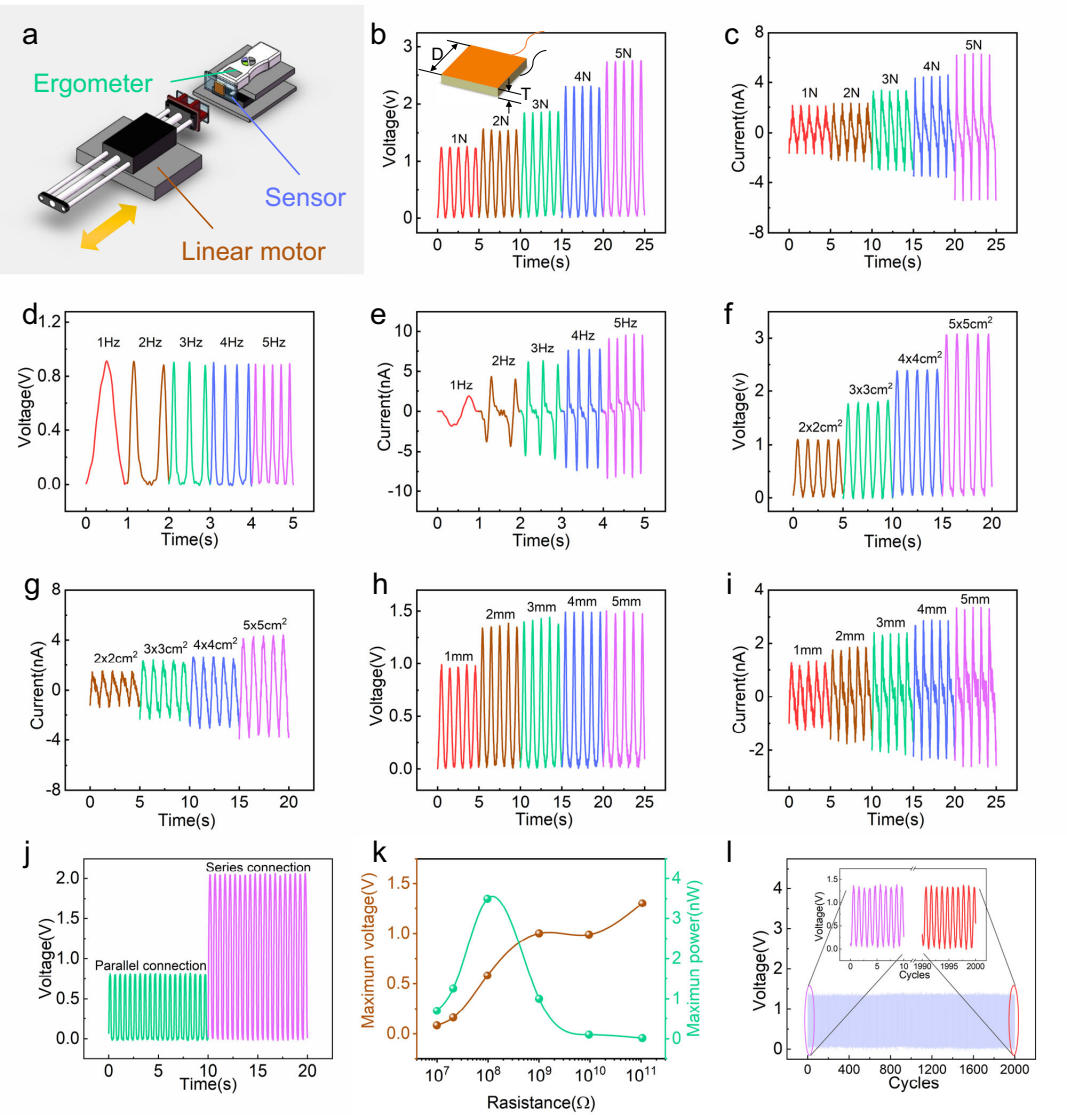
Electrical characteristics of the TENG sensors. **a** Platform with adjustable pressing force and frequency consists of a linear motor, ergometer and sensor. **b**, **c** The open-circuit voltage and short-circuit current obtained by pressing the sensor at 1 Hz frequency with different forces (from 1 N to 5 N). Inset: schematic diagram of the sensor size. **d**, **e** The open-circuit voltage and short-circuit current output of the sensor at the pressing frequency (from 1 Hz to 5 Hz) with a force of 5 N. **f**, **g** Open-circuit voltage and short-circuit current output obtained from sensors with different areas at a frequency of 1 Hz with a force of 5 N. **h**, **i** The open-circuit voltage and short-circuit current output obtained from sensors with different thicknesses at a frequency of 1 Hz with a force of 5 N. **j** The open-circuit voltage for two sensors placed in series and in parallel. **k** The maximum output power and maximum voltage curves for the triboelectric sensor used as a power supply with different external load resistances (ranging from 10^7^ to 10^11^ Ω) with a force of 5 N at a frequency of 2 Hz. **l** Mechanical durability test for up to 2000 press-release cycles. Inset: the voltage signals generated for the initial 10 s and the final 10 s.

The electrical characteristics of the triboelectric sensor are crucial for lip reading. The influences of parameters such as force, frequency, sensor size, series and parallel structure on electrical signals were investigated. The output voltages and powers with various external load resistances and durations were studied. The experimental platform including the linear motor and ergometer is shown in Fig. 2a. The sensor fixed onto the ergometer was pushed periodically by a linear motor at a given frequency. The pushing forces were varied by adjusting the spacing. The sensor is a square with length *D* and thickness *T*, as shown in the inset of Fig. 2b. Electrical signals generated by various materials (Paper/ Polyethylene terephthalate (PET)/ Poly tetra fluoroethylene (PTFE)/ Polyimide (PI) / Polyvinyl chloride (PVC)/ Fluorinated ethylene propylene (FEP)) contact nylon were recorded using a 20 × 20 × 5 mm^3^ sensor with a force of 5 N at a frequency of 2 Hz, as shown in Supplementary Fig. 2a. Both PVC and FEP show better output performance with nylon, and PVC is chosen for its better toughness. The open-circuit voltage and short-circuit current curves are shown in Supplementary Fig. 2b, c.

Electrical signals generated with a variable force at different frequencies are shown in Fig. 2b–e. Voltages and currents obtained using a 20 × 20 × 5 mm^3^ structure with variable forces (from 1 N to 5 N) at 1 Hz are shown in Fig. 2b, c. The sensor is sensitive at the pressure above 1 N, and the sensitivity is 0.376 V/N. Voltages and currents obtained using a 20 × 20 × 5 mm^3^ structure with 5 N forces at various frequencies (from 1 Hz to 5 Hz) are shown in Fig. 2d, e. The current increases with increasing frequency, from 1.88 nA to 9.66 nA, while the output voltage signal basically remains the same, and the voltage fluctuation is <0.02 V (0.89–0.91 V).

Voltages and currents obtained with 5 N forces at 1 Hz using a *D* × *D* × 2 mm^3^ (variable length *D* from 20 mm to 50 mm) structure are shown in Fig. 2f, g. The output voltage and current increase with the area, the voltage increases from 1.10 V to 3.08 V, and the current increases from 1.52 nA to 4.39 nA. The larger the contact area, the more charges generate and transfer between the electrodes, resulting in a greater current output. The voltage increases with the contact area due to the increased friction produced by the sensor structure. For better output, the area of the sensor needs to be increased. However, the sensor area is restrained by the size of the muscle being monitored. Voltages and currents obtained with a force of 5 N at 1 Hz using a 20 × 20 × *T* mm^3^ (variable thickness *T* from 1 mm to 5 mm) structure are shown in Fig. 2h, i. The output voltage and current increase with thickness until the thickness *T* reach 2 mm, and then the output voltage and current barely change. The electrostatic induction of the charge decreases hyperbolically with increasing spacing; when the thickness reaches 2 mm, the electrostatic induction becomes weak, with only a little charge increase on the electrodes.

The series and parallel connection of the two sensors are investigated in Fig. 2j. Voltage signals in series are stronger than those in parallel due to the greater area in the series connection. Current signals in series and parallel are shown in Supplementary Fig. 2d. The curves for power and voltage with different external load resistances are shown in Fig. 2k. The maximum output power of the sensor is 3.50 nW with an external resistance of 97.1 MΩ, which indicates that the internal resistance of the sensor is ~100 MΩ. The current curve with an external load is shown in Supplementary Fig. 2e. The signal duration of the sensor changes with the number of presses, as shown in Fig. 2l. Under the periodic pressing of a 5 N force at 2 Hz, the output voltage of the 20 × 20 × 2 mm^3^ sensor is basically attenuated less (from 1.40 V in the beginning to 1.38 V at the end) after 2000 cycles of continuous pressing, indicating a good voltage signal duration.

The impact of artificial sweat (Artificial sweat, PH5.5, CF-001, Chuangfeng Co., LTD) on the sensor’s performance is shown in Supplementary Fig. 2f, g. The open-circuit voltage and short-circuit current are measured with 0–4 drops (0.0157 ml/drop). The result shows that the artificial sweat has little effect on the electrical output performance of the sensor. The comparison of this triboelectric sensor with capacitive, piezoresistive and piezoelectric sensors are discussed in Supplementary note 3.

### Electrical signals generated by mouth motion and their comparison with sound signals

**Fig. 3:**
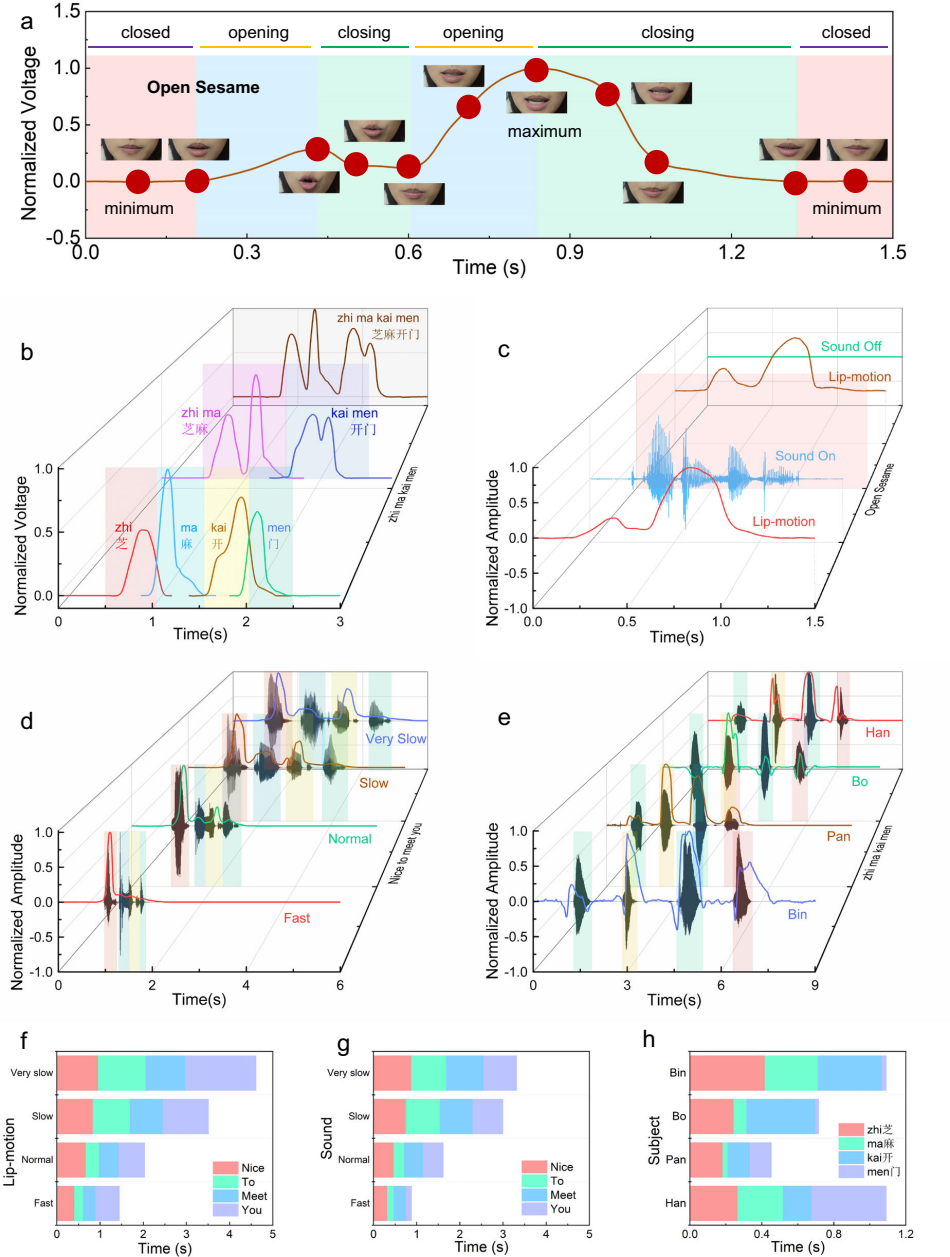
Signals generated by mouth muscles and a comparison of sound and lip-motion signals. **a** In a typical speaking sequence (“Open Sesame”), the mouth shape synchronizes with the signal, and the regions separated by the mouth state are denoted as closed, opening and closing. **b** The combined and decomposed lip-motion signals for “Zhi”, “Ma”, “Kai”, “Men”, “Zhi Ma”, “Kai Men”, and “Zhi Ma Kai Men”. **c** The lip-motion signals for silent and vocal speaking remain the same, and sounds are recorded synchronously when speaking “Open Sesame”. **d** Sound and lip-motion signals are collected simultaneously at four speeds of reading “Nice To Meet You”. **e** Sound and lip-motion signals collected simultaneously when four participants (Han, Bo, Pan and Bin) read “Zhi Ma Kai Men”. **f** The time used for lip-motion signals for each word in “Nice To Meet You” at four different reading speeds. **g** The time used for sound signals for each word in “Nice To Meet You” at four different reading speeds. **h** The time statistics for the lip-motion signals ahead of the sound signals for each word in “Zhi Ma Kai Men” spoken by four participants.

The mouth shape signal was detected during normal vocalization with the manufactured sensor, as shown in Fig. 3. Two types of signals are mainly observed: one is the waveform signal corresponding to the pronunciation of vowels, and the other is the waveform signal for words and phrases. To highlight the difference in signal waveforms, the amplitude interference is removed by normalization.

The corresponding relationship between the sensor signals and the mouth shapes in detail is shown in Fig. 3. Taking speaking “Open Sesame” as an example, the lip-motion signals and video are collected simultaneously for 1.5 s. The recorded lip-motion signals are normalized. Three typical shape types in the signal curve correspond to the closed, opening, and closing-mouth states. The signal curve shape divides the time region into eight parts, including two closed-mouth parts, two opening-mouth parts and four closing-mouth parts. There are two peaks in the curve: “Open” corresponds to a small peak, and “sesame” corresponds to a large peak. Vowels have a larger influence on mouth movements than consonants, and 12 vowel signals and corresponding mouth shapes are collected in Supplementary Fig. 5a–l. The lip-motion signals when pronouncing /a:/ with different mouth-opening sizes are shown in Supplementary Fig. 6a.

To understand the differences and similarities of the pronunciation between phrase and words in the phrase, the lip signals corresponding to the pronunciation of words and phrase were captured, as shown in Fig. 3b. Take the Chinese phrase “zhi ma kai men” for example. The Chinese phrases can be disassembled into Hanzi combinations, as can signals captured. There are 3 rows: the front, middle and back rows corresponding to different combinations in Fig. 3b. The back row shows the consecutive lip-motion signals for the Chinese phrase “zhi ma kai men”, while the middle row shows the lip-motion signals for the Chinese phrase combination: “zhi ma” and “kai men” one by one, and the front row shows lip-motion signals for the Hanzi characters: “zhi”, “ma”, “kai”, and “men” one by one. Signal waveforms in the front and middle rows are assembled the same as the signal waveform in the back row. To express information faster, humans automatically omit some parts of pronunciation, and this feature can be clearly observed from the signal difference in Fig. 3 b. The lip signal corresponding to a single word in the phrase is shorter, and the signal duration efficiency is improved. For “zhi ma kai men”, the efficiency of continuous signals in the back row (1.648 s) is 31.3% higher than that of single-Hanzi signal combinations in the front row (total 2.398 s). For “open sesame”, it takes 1.10 s to continuously read the phrase and a total of 1.286 s to read two words separately, which increases the efficiency by 14.5%. More details are stated in Supplementary Fig. 6b. The data manipulation process is demonstrated in Supplementary note 6.

The sound and lip motion are related naturally when speaking. The lip-motion signals and the sound signals are synchronously collected and compared, and the relationship between the signals is studied. Low-pass filtering is used to remove power-frequency interference signals with a cut-off frequency of 20 Hz. To remove the interference in the waveform amplitudes caused by different conditions, the amplitudes of the signals are normalized, as shown in Fig. 3c–e.

The lip-motion signals and sound signals for “open sesame” were collected in the sound-on-lip-on and sound-off-lip-on modes, distinguished by a grey surface, as shown in Fig. 3c. The sound-on-lip-on mode is listed in the front, and the sound-off-lip-on mode is listed in the back. The lip-motion signals have good consistency in the sound-on and sound-off modes. The vocalization does not twist the lip-motion signal waveform. This phenomenon is useful for people with throat injuries to communicate with lip language. In addition, the data volume for lip-motion signals (12 KB) is much smaller than that for sound signals (576 KB), only 1/48, which means that the data acquisition, storage and transmission of lip-motion signals consumes fewer resources.

The influence of speaking speed on lip-motion signals is studied. Figure 3d shows the difference between the lip-motion signal and the sound signal of the phrase “nice to meet you” collected simultaneously for one person at different speech speeds. The start and endpoints of the sound are easy to identify and are used as to identify the phrase length, and the phrase durations are obtained, as shown in Fig. 3d. There are four durations based on sound: very slow (3.988 s), slow (3.383 s), normal (1.636 s) and fast (0.857 s). The signal waveforms are basically similar at different speech speeds, but the relative amplitude of the lip movements at the end of the phrase changes. The relative amplitudes of the signal “meet” are shown in Fig. 3d, and the normalized peak values are 0.6725, 0.4922, 0.3170, and 0.0879.

Figure 3e shows the difference in lip motion and sound signals collected from four persons (Pan, Bo, Bin and Han) when they pronounce the phrase “zhi ma kai men”. Both lip-motion signals for Bo and Bin show slight downwards troughs at the beginning of each word, but the signals for Pan and Han do not show this feature. In addition, the four participants showed obvious differences in speech speed. The differences in these signal curves are due to the unique speaking and pronunciation habits formed for each people over many years. Different muscle stretching habits lead to significant individual variation in the details for the lip-motion signals of people, indicating potential for use in applications for identification.

The duration of the lip-motion signal is longer than the duration of the sound signal, as shown in Fig. 3f, g. The phrase durations for the sound signals account for 60.8%-85.4% of the lip-motion signal durations. Each word exhibits different proportions at different speech speeds for continuous reading and omission of syllables. The proportion of words has a strong relationship with the number of syllables. The proportions of polysyllabic words increase with speech speed. The proportion of “nice” sound signals increases from 26.5% to 36.2%, and the proportion of lip signals increases from 20.7% to 28.1%; the proportion of “meet” sounds increases from 26.0% to 32.3%, and the proportion of lip signals changes from 19.8% to 19.9%. Meanwhile, the proportion of one-syllable words decreases with speech speed. The proportion of “to” sound signals decreases from 24.4% to 16.8%, and the lip signals decrease from 23.9% to 14.0%; the proportion of “you” sound signals decreases from 23.1% to 14.8%, and the lip signals change from 35.5% to 38.0%. One-syllable words are much more compressed than polysyllabic words in fast speech in both sound and lip signals.

The lip-motion signal starts earlier than the sound signal because people open their mouth firstly and then speak. Figure 3h counts the time advances for the lip-motion signal relative to the sound signal when four persons (Pan, Bo, Bin, and Han) say the phrase “zhi ma kai men”, and the overall time advance is ~18–417 ms. There are individual variations in the time advance due to the habits of the speakers. The first syllable of “zhi” and “kai” are /zh/ and /k/, respectively, which are open sounds, so the time advance is larger, between 124–417 ms; the first syllable of “ma” and “men” /m / is a nasal sound, which does not require the opening of the mouth, and the time advance is small, 18–295 ms, as shown by the data obtained for Pan, Bo, and Bin. Han has obvious lip motions when pronouncing /m/, leading to a larger time advance, as shown in Han’s data.

### Neural network classifier trained for lip language recognition

**Fig. 4:**
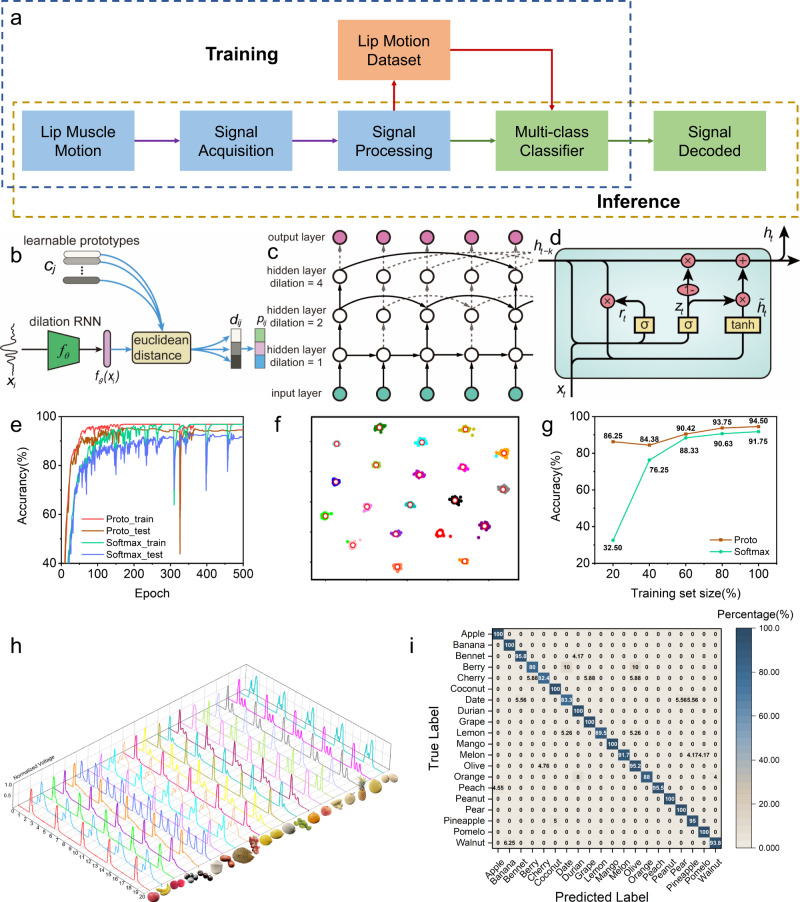
A signal classification experiment based on deep learning. **a** Deep learning aided data process flow, including a training and an inference process. **b** Schematic diagram of the overall structure of the dilated recurrent neural network model based on prototype learning. **c** Structure diagram of the feature extractor in the model. **d** The basic unit of the dilated recurrent neural network GRU. **e** The training and testing accuracy curves obtained during the learning process for the dilated recurrent neural network based on the softmax-classification layer and prototype learning. **f** Visualization of the two-dimensional features of the dilated recurrent neural network based on prototype learning. **g** Comparison of the test accuracy for two models with different sample sizes. **h** 3D plot of the lip-motion signals generated for 20 spoken words (fruit) in the dataset. **i** The confusion matrix for lip-motion signals for 20 words (fruits).

A typical scenario of lip language is to help people with speech impairment communicate without barriers. For people with speech impairments, a lip-language recognition system helps them quickly interpret lip motion through algorithms and finally achieve barrier-free communication with normal people through sound and light media. Considering the frequency and complexity of daily communication and the distinguishability of the lip-motion signals detected by the sensor, it is necessary to use deep learning algorithms to accomplish the intelligent recognition of lip language. LLDS contains two data flow processes: training and inference, as shown in Fig. 4a. In the training stage, the dataset that consists of lip-motion signals is used to train the deep learning model. At the inference stage, new signals are decoded by the trained model for recognition. However, deep learning models are typically data hungry, which presents a major challenge for training a high-accuracy model with limited lip-motion samples.

To address the above issue, we propose a dilated recurrent neural network (dilated RNN) model^49^ based on a prototype learning approach^50^, and more details are shown in Supplementary note 7. Figure 4b illustrates the framework of our lip signal recognition method in this study. For each category, the model learns a prototype in the deep feature space. In the classification stage, signals are classified by prototype matching. Specifically, the training sample passes through the feature extractor to obtain its representation in the deep feature space. By calculating the Euclidean distance between the feature vector of the sample and the category prototypes, the category to which it belongs is obtained. Figure 4c shows a schematic diagram of the structure of the feature extractor in the model. The feature extractor is a multilayer dilated recurrent neural network. The recurrent neural network helps capture long-term dependencies in the sequence. The dilated mechanism effectively reduces the number of model parameters and significantly improves the training efficiency. In particular, the gated recurrent unit (GRU)^51^ is chosen as the basic unit of the recurrent neural network, including the update gate and reset gate used to capture the dependencies in the sequence. The structure is shown in Fig. 4d. The data collection process is described in Supplementary note 8. Data preprocessing for machine learning is demonstrated in Supplementary note 9.

To facilitate comparison, we use the softmax-classification layer and prototype learning in dilated RNN to classify the collected signals. The experimental data contain 20 categories (20 fruit names are selected in Supplementary Fig. 7a–t, and lip-motion signals are collected in Supplementary Fig. 8a–t). Each category contains 100 training samples, among which 80 are used for training and 20 are used for testing. A 4-layer dilated recurrent neural network with 50 neurons in each layer is adopted. Figure 4e shows the training accuracy and testing accuracy for the learning process for the two models. After training for 500 epochs, the test accuracy of the dilated RNN with the softmax-classification layer reaches 91.75%, and the test accuracy of the prototype learning reaches 94.50%, which is significantly higher than that of the model based on the softmax-classification layer. In addition, it can be observed from the accuracy curve in the training process that the model based on prototype learning converges faster than the model of the softmax-classification layer.

The 2D feature space is selected to visualize the distribution of samples in the feature space. Figure 4f shows the distribution of training samples in the feature space for the prototype learning models (for the distribution of training samples in the softmax-classification-layer model, see Supplementary Fig. 9a). Different colours represent different categories. The distribution of samples in the feature space with the prototype learning model shows stronger interclass separability and intraclass compactness than the softmax model.

In particular, small sample classification experiments were carried out. The test accuracy curves for the models based on prototype learning and softmax for a training sample number gradually decreased to 100/80/60/40/20% of the original number are shown in Supplementary Fig. 9b, c. Figure 4g compares the accuracy of the two models with different sample sizes. The model based on prototype learning has a significantly better performance on the test accuracy than the softmax model when there are fewer training samples. When the training data are 20%, the model based on prototype learning has a test accuracy of 85.23%, while the model based on softmax only has a test accuracy of 31.46%; the former is ~2.7 times the latter. The impact of hyperparameters, including the number of layers and neurons in each layer, on the test accuracy is shown in Supplementary Fig. 9d–f.

Figure 4h shows the different lip-motion signals for 20 words. Each word repeated five times in each lip-motion signal line gives good repeatability, and the intercepted signal line lasts for 20 sec. The lip motion for most words shows significantly unique waveforms, while some words show similar waveforms, such as Berry and Olive. The detailed confusion probabilities for the 20 words’ lip-motion signals based on prototype learning are shown in the confusion matrix in Fig. 4i. The much closer signals have a higher confusion probability. The total recognition accuracy of the current model is 95%, while each word has a different recognition accuracy on the diagonal. Forty-five per cent of words have 100% recognition accuracy, 80% of words have >90% accuracy, and 100% of words have >80% accuracy. Some confusion happens; for example, with 10% probability, Berry is recognized as Date or Olive. Words with similar lip-motion signals can be searched out with the help of a confusion matrix and guide further improvement of the sensor. The detailed confusion probabilities for 20 words’ lip-motion signals based on softmax are shown in Supplementary Fig. 10a, and a comparison of the loss function curves is shown in Supplementary Fig. 10b.

### Applications of the lip-language decoding system

The lip-motion signals detected by the sensors are collected and prepared for the development of applications, such as identity authentication to unlock gates and control the direction of car motion by lip-motion and providing assistances with communication for people lacking a voice.

**Fig. 5:**
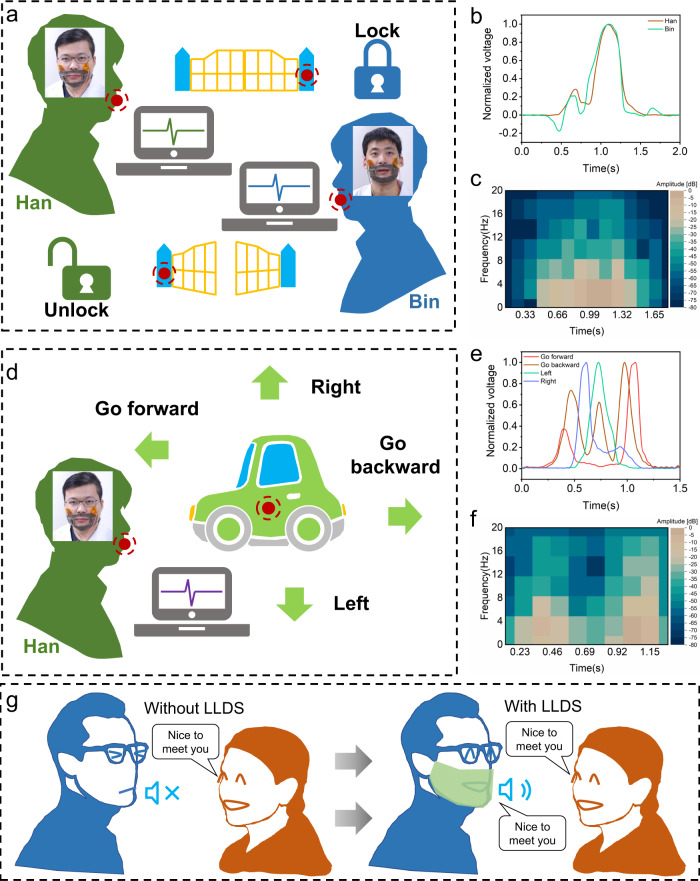
The applications for lip-language decoding in personal identity verification (PIV), toy-car control and lip motion to speech conversion for assisting with communication for people lacking a voice. **a** Schematic diagram of unlocking a gate by lip motion with personal identity verification. **b** A comparison of lip-motion signals from participants (Han and Bin) in the time domain. **c** Short-time Fourier transform (STFT) analysis of the lip-motion signals from Han. **d** Schematic diagram of direction control for toy car motion by lip motion. **e** Comparison of lip-motion signals from Han in the time domain. **f** STFT analysis of the lip-motion signals for ‘Go forwards’ from Han. **g** Schematic diagram of the daily voice communication for people lacking a voice with and without lip-language decoding system (LLDS).

Figure 5a shows the schematic process for unlocking a door by lip-motion signal recognition. When the host (Han) speaks out the unlock instruction (“Open Sesame”), the system recognizes the host’s lip signal and the door opens; when the guest (Bin) speaks out the same unlock instruction, the lip-motion signal identification fails and the door remains closed. This application demonstrates the ability of personal identity verification by lip-motion recognition. The lip-motion signals of the host and guest in the time domain (shown in Fig. 5b) and in the frequency domain (in Supplementary Fig. 11a) are compared. Short-time Fourier transform (STFT) graphs for the lip-motion signals from Han and Bin are compared in Fig. 5c and Supplementary Fig. 11b. The normalized voltage waveform and amplitude of Han’s signals are all clearly distinguishable from that of Bin’s and can be used as discrimination criteria. See Supplementary Movie 1 for the video.

Figure 5d shows the application process for controlling the movement direction of a toy car with lip-motion signal recognition, which demonstrates the lip-motion recognition ability of distinguishing instructions from one person. There were four directional instructions: “Go forwards”, “Go backwards”, “Left” and “Right”. Figure 5e shows the time-domain curves for the lip motion for instructions sent by Han to control the four directions of movement for a car. The corresponding frequency-domain curves are shown in Supplementary Fig. 11c. There are some differences in the normalized voltage amplitudes for the four instructions from Han. The waveforms of the four instructions are significantly different. STFT graphs for the forward instructions from Han are shown in Fig. 5f, and graphs for the backward, left and right instructions are shown in Supplementary Fig. 11d–f. Lip-motion signals for the instructions from one person show a difference and can be recognized and used for direction control. See Supplementary Movie 2 for the video.

Figure 5g shows a possible scenario for daily voice communication with friends for a person lacking a voice with and without LLDS, which demonstrates the possibility of using LLDS in helping voice-missing persons regain the ability of daily voice communication. A person missing a voice cannot respond to greetings from friends in natural voice. However, with the assistance of LLDS, such a person is able to respond to a friend with lip motions, which are captured, analysed, decoded into specific expressions (words or sentences) and translated into voice and text. In this application, the participant (Han) speaks out phrases with the voice-off, LLDS captures the related lip-motion signals, completes the recognition and invokes the voice play. See Supplementary Movie 3 for the video. With such technology, people lacking a voice can enjoy ordinary daily life with no barrier in voice communication, which helps boost their happiness and offers great convenience in conversation.

## Discussion

In this study, we proposed and investigated a lip-language interpretation system based on triboelectric sensors operated in contact-separation mode. The triboelectric sensors attach to the lip muscles and capture lip motion and transfer the measured electrical signals to a decoding system, which are then translated into a communication language. The principles and mechanical and electrical properties of the TENG sensor were tested and analysed. Signal patterns for selected vowels and words were collected, and signal characteristics analysed. The lip and sound signals were compared simultaneously, proving that the lip motion for silent speech equals that of voiced speech. The impact of different parameters, such as speaking speed and lip-motion pattern, on collected signals was analysed. A dilated recurrent neural network model based on prototype learning is proposed and employed in lip language recognition, which achieves a test accuracy of 94.5% for the case of 20 classifications and 100 samples per classification. Applications based on the lip-language interpretation system with triboelectric sensors, such as identity recognition to open a door, directional control of a moving toy car and voice translation, work well and show feasibility and potential for lip-language recognition. This enriches the ways and means of helping people with speech disorders live a convenient life with barrier-free communication. This work has potential value in applications involving robot control, personal identity verification, human-machine interfaces, disability assistance, silent speech, intelligence, anti-terrorism mission implementation, rehabilitation, biomedical engineering and VR.

## Methods

### Fabrication of sensors

The preparation of TENG sensors can be divided into electrode preparation and assembly. In the TENG sensor, nylon and PVC were used as friction materials, and a copper foil was used as the electrode material. Square sheets with a side length of 40 mm were cut from the purchased nylon (0.08 mm) and PVC (0.08 mm) films. A square copper foil with a side length of 30 mm was attached to the diaphragm as electrodes. Copper wires with a diameter of 0.71 mm were soldered onto the copper foil. Polyurethane sponge was used as a spacer material; a 3.5 mm thick polyurethane sponge was cut into a square block with a side length of 40 mm, and a square block was cut out with a side length of 20 mm in the middle to make a spacer block 10 mm wide on each side. The two electrodes were fixed onto both sides of the gap, and PI material was used to package the sensor as a whole.

### Mask Fabrication

The mask was prepared using nonwoven fabric, with a folded structure in the middle and exposed lip area to minimize the impact on normal speech. The mask was fixed onto the face by three rubber bands at the top, middle and bottom. Two triboelectric sensors were installed at the left and right corners of the mouth (corresponding to the orbicularis oris muscle) to capture the movement signals for the orbicularis oris muscle. The two sensors are connected in series to increase the signal amplitude.

### Fabrication of the hardware used for lip motion activated gate unlocking

Geometric models (Solidworks) of two gates and gate frames were 3D printed using PLA materials. The lip-motion signals collected by the mask were analysed by software to obtain control signals, and the control signals were transferred to a single-chip microcomputer through conversion (Wison CH340G USB to TTL module) (STM32F103C8T6 small system version). Then, the steering gear (SG90 steering gear) was controlled to open the door.

### Fabrication of the hardware used for lip motion activated remote control of a toy car

The purchased modules were assembled into the toy car. The lip-motion signal collected by the mask was analysed by the software to obtain the control signal and transmitted to a Bluetooth module on the car (Vision BT08B Bluetooth serial module). The signal was transmitted to the main board (Arduino UNO), and the rotation of the motor (1:48 strong magnetic anti-interference carbon brush motor) was controlled to execute the forward, backward and steering tasks of the car.

### Measurement of force and electric characteristics

In the output performance experiment for the TENG sensor, a linear motor (LinMot P01-37×120-C_C1100) was used to load cyclic pressure, and a dynamometer (PUYAN DS2) was used to measure the pressure in the experiment in real time to ensure the uniformity of the experimental conditions. In this study, an NI 9215 acquisition card was used to collect data. A 6514 electrometer was used to measure the voltage, current and other signals. The software platform was built based on LabVIEW for real-time acquisition, analysis and processing of the experimental data. A microphone was used to collect real-time voice signals when speaking for the corresponding analysis.

### Reporting summary

Further information on research design is available in the Nature Research Reporting Summary linked to this article.

## Supplementary information

Supplementary Information

Description of Additional Supplementary Files

Supplementary Movie 1

Supplementary Movie 2

Supplementary Movie 3

Reporting Summary

## Data Availability

All experimental data generated in this study are available and presented in the paper and the Supplementary Information. Source data are provided with this paper.

## Source data

Source Data
