# Supplementary material for: Decoding lip language using triboelectric sensors with deep learning
Source: Nat Commun. 2022 Mar 17;13:1401. doi: 10.1038/s41467-022-29083-0 (PMC8931018; doi:10.1038/s41467-022-29083-0)
Supplement: Supplementary file 1 — Supplementary Information [file 41467_2022_29083_MOESM1_ESM.pdf]

Supplementary Information

Decoding lip language using triboelectric sensors with deep learning

Yijia Lu[1†], Han Tian[1†], Jia Cheng[1†], Fei Zhu[2], Bin Liu[1], Shanshan Wei[1], Linhong Ji[1], Zhong Lin Wang[3,4,5★]

[1] State Key Laboratory of Tribology, Department of Mechanical Engineering, Tsinghua University, Beijing 100084, China.

[2] National Laboratory of Pattern Recognition, Institute of Automation, Chinese Academy of Sciences, Beijing, 100190, China.

[3] Beijing Institute of Nanoenergy and Nanosystems, Chinese Academy of Sciences, Beijing 101400, China.

[4] School of Nanoscience and Technology, University of Chinese Academy of Sciences, Beijing 100049, China

[5] School of Materials Science and Engineering, Georgia Institute of Technology, Atlanta, GA 30332-0245, USA

★email: zhong.wang@mse.gatech.edu

Supplementary Information

Supplementary note 1: the simulation results ... 3

Supplementary note 2: electrical characteristics of sensors. ... 3

Supplementary note 3: comparison of the sensors (Piezoresistive, Capacitive, Piezoelectric and Triboelectric sensors). ... 4

Supplementary note 4: The normalized waveform of main vowels ... 7

Supplementary note 5: the characteristics of lip motion signals ... 8

Supplementary note 6: Voltage data manipulation ... 8

Supplementary note 7: setup of the neural network classifier. ... 8

Supplementary note 8: Data collection process ... 9

Supplementary note 9: Data preprocessing for machine learning ... 9

Supplementary note 10: the chosen words and phrases ... 10

Supplementary note 11： Waveform of the 20 fruit words ... 12

Supplementary note 12: impact of hyper-parameters of dilated RNN ... 13

Supplementary note 13: training results of the dilated RNN ... 13

Supplementary note 14: analysis of the lip-motion signals. ... 14

Supplementary References ... 14

Supplementary note 1: the simulation results

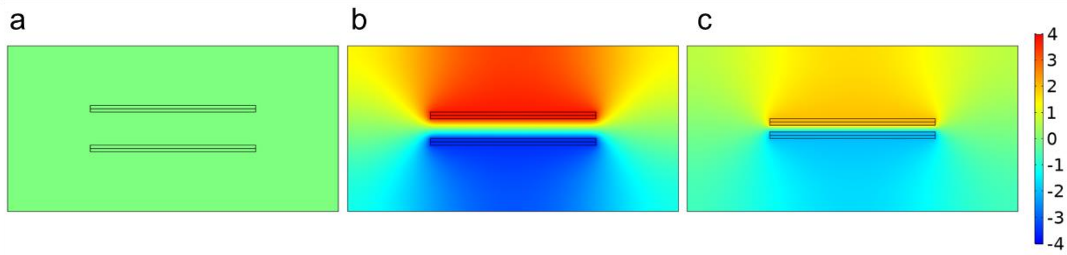

Supplementary Fig. 1 The spatial distribution of electric field at different stages (Simulated by COMSOL Multiphysics). (a) Initial or released stage. (b) Pressing or releasing stage. (c) Maximum pressed stage.

Supplementary note 2: electrical characteristics of sensors.

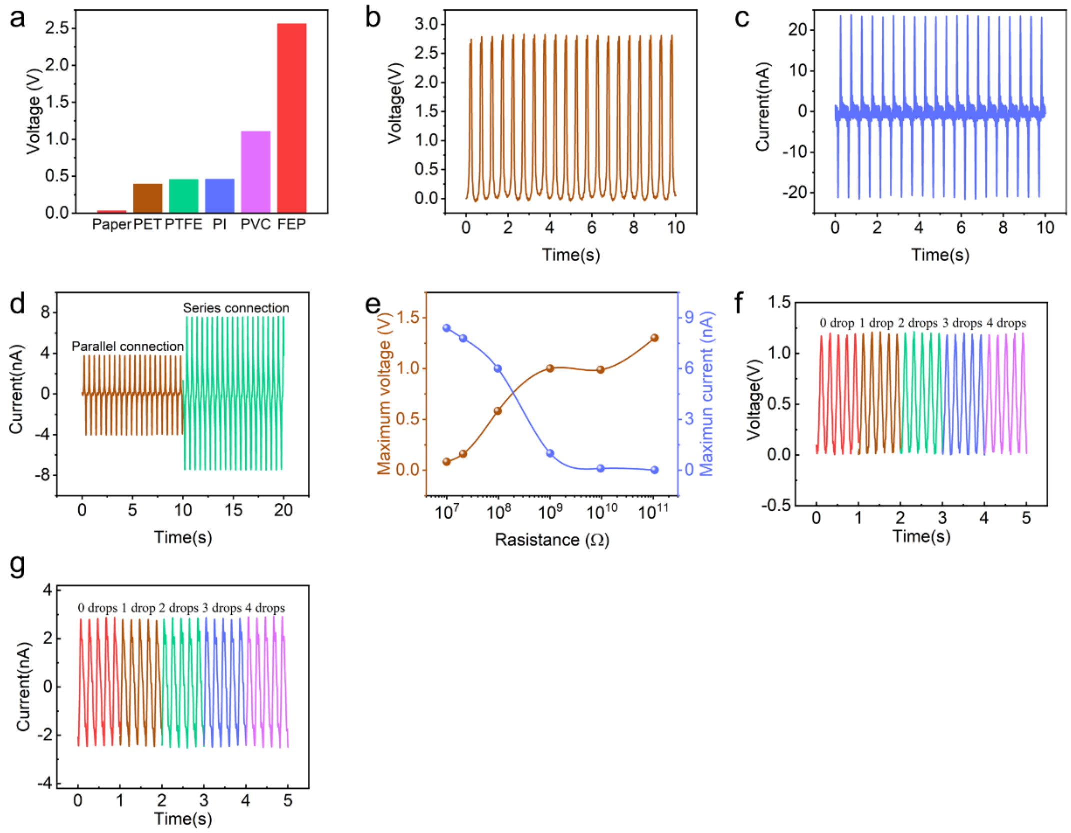

Supplementary Fig. 2 Electrical characteristics of sensors. (a) The output open circuit voltage of 5 different materials friction with nylon. (b-c) The open circuit voltage (Voc) and short circuit current (Isc) of the triboelectric sensor as a power supply (5 N force pressing at 2 Hz frequency). (d)The short circuit current of two sensors in series and parallel respectively. (e) The effective

value curve of the output voltage and current of the TENG under different loads. (f-g) The open circuit voltage (Voc) and short circuit current (Isc) of the triboelectric sensor with different drops of artificial sweat (pressed with a force of 5 N at a frequency of 5 Hz).

Supplementary note 3: comparison of the sensors (Piezoresistive, Capacitive, Piezoelectric and Triboelectric sensors).

Piezoresistive, piezoelectric, capacitive and triboelectric sensors are all electromechanical sensors which transduce the applied force into electrical signals via different mechanisms. They can be divided into two groups: group A includes piezoresistive and capacitive passive sensors, group B includes piezoelectric and triboelectric active sensors.

The resistance or capacitance changes when the sensors in group A is pressed by force. The force of compression can be obtained from the change of resistance or capacitance. In order to measure the change of resistance or capacitance, an additional voltage is needed, and the change of resistance or capacitance is obtained through the change of current. Electrical signals generated by pressure or contact cannot be measured without an additional voltage.

Piezoelectric and triboelectric sensors in group B are both self-powered sensors. Piezoelectric sensors make use of the piezoelectric effect, which creates a voltage when charge accumulates on both sides of the material during compression and deformation. The triboelectric sensors make use of two effects: contact electrification and electrostatic induction. The friction between two interfaces with different surface energy generates charges, which will further induce charges on the adjacent electrodes. Change of the distance between two interfaces will cause the change of the amount of induced charges, and will produce instantaneous voltage and current in the external circuit. Electrical signals generated by pressure or contact can be measured without an additional voltage, which is called self-powered.

Comparing to group A, the self-powered characteristic of group B means low-power consumption, which is of great importance for small-scale wearable electronics (sensors and devices) and low carbon living. With the booming of the Internet of Things (IoT), numerous lightweight and wearable sensors have been developed for biomedical monitoring. In the case of a certain battery capacity, low energy consumption can prolong the working time of the device and reduce the charging frequency. In addition, group B does not require additional circuit design and power supply to generate electrical signals, whereas group A does.

Piezoelectric generators (PG) and Triboelectric nanogenerators (TENG) are two most common approaches for energy harvesting in group B. The two generators are compared (Ahmed et al[1], 2020) at frequency values below 4 Hz, which is typical of human motions. TENG shows higher power performance and is almost independent of the operating frequency, making it highly efficient comparing to PG. Low cost is another advantage of TENG. Various thin film materials which are common in daily life, e.g. paper, can be used to fabricate the TENGs, which shall greatly lower the cost.

Sensitivity is a key parameter of sensors. There are a variety of sensitivities of different types of sensors in literatures. The sensitivities of different sensors in literatures are shown in Supplementary Tab. 1.

Reference	Materials	Electrode	Sensitivity	Mechanism
This work	PET/ nylon	Cu foil	0.61183 V/kPa / 0.376 V/N /0.514 kPa^{-1}	Triboelectric
**Wu** et al[2], 2016	PDMS/ CB/ PU fiber		0.068 kPa^{-1}	Piezoresistive
**Lou** et al[3], 2017	rGO-textile		0.012 kPa^{-1}	Piezoresistive
**Kim** et al[4], 2019	3D spacer textile/ PUD /CNT ink		0.03-0.31 kPa^{-1}	Piezoresistive
**Liu** et al[5], 2019	ionogel infiltrated paper		0.304 kPa^{-1}	Piezoresistive
**Park** et al[6], 2017	Porous Ecoflex	AgNW/ carbon fiber film	0.161 kPa^{-1}	Capacitive
**Sheng** et al[7], 2016	Polyethylene	Liguid GalnSn alloy	0.17 kPa^{-1}	Capacitive
**Joseph** et al[8], 2017	Silk	Au	0.00326 V/kPa	Piezoelectric
**Ghosh** et al[9], 2017	Fishskin collagen		0.027 V/N	Piezoelectric
**Guo** et al[10], 2018	PVDF/ BaTiO3 NW	Al/ Cu foil	0.017 kPa^{-1}	Piezoelectric
**Pu** et al[11], 2017	PDMS, 3M VHB	PAAM-LiCl hydrogel	0.013 V/kPa	Triboelectric
**Rasel** et al[12], 2018	PDMS/PDMS-MWCNT	Au	0.51 V/kPa	Triboelectric
**Liu** et al[5], 2019	Ionogel infiltrated Paper / Paper	IIPFE	0.0206 V/N	Triboelectric

Supplementary Tab. 1 Sensitivity, materials and electrodes of different sensors in literature

Liu et al[5], 2019 developed an ionogel infiltrated paper based flexible electrode and fabricated two kinds of all-paper based sensors. By comparing the piezoresistive and triboelectric sensors, he found that TENG sensor have a broader measuring rang (0.45 - 6.5 N) and higher sensitivity (20.6 mV/N) than piezoresistive sensor (0.304 within the range of 0.3 - 0.9 N).

TENG-based sensors have advantages of low-power consumption, low cost, various choice of materials and easy fabrication.

The capacitive (ESPB-01, RENHE Co. LTD), piezoresistive (DF9-16, CHENGTec Co. LTD), piezoelectric (LDT0-028K, piezoelectric Polyvinylidene Fluoride, TE) and our triboelectric sensors are collected to make the comparison, as shown in Supplementary Fig. 3. The sensitivities of these sensors are measured and analyzed, as shown in Supplementary Fig. 4. The sensitivity, trigger point and cost of each sensor are compared in Supplementary Tab. 2. Our triboelectric sensor has the lowest cost and the highest sensitivity in the four sensors.

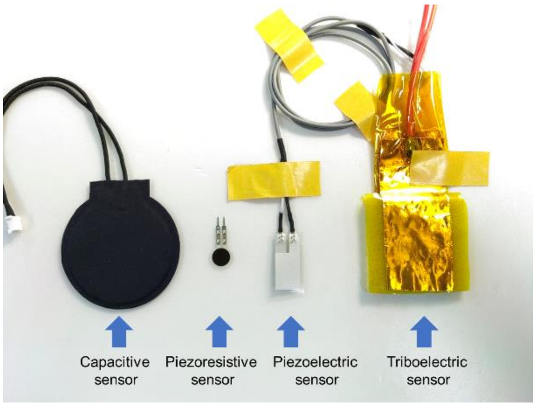

Supplementary Fig. 3 The sensors for testing, from left to right are capacitive, piezoresistive, piezoelectric and triboelectric sensors.

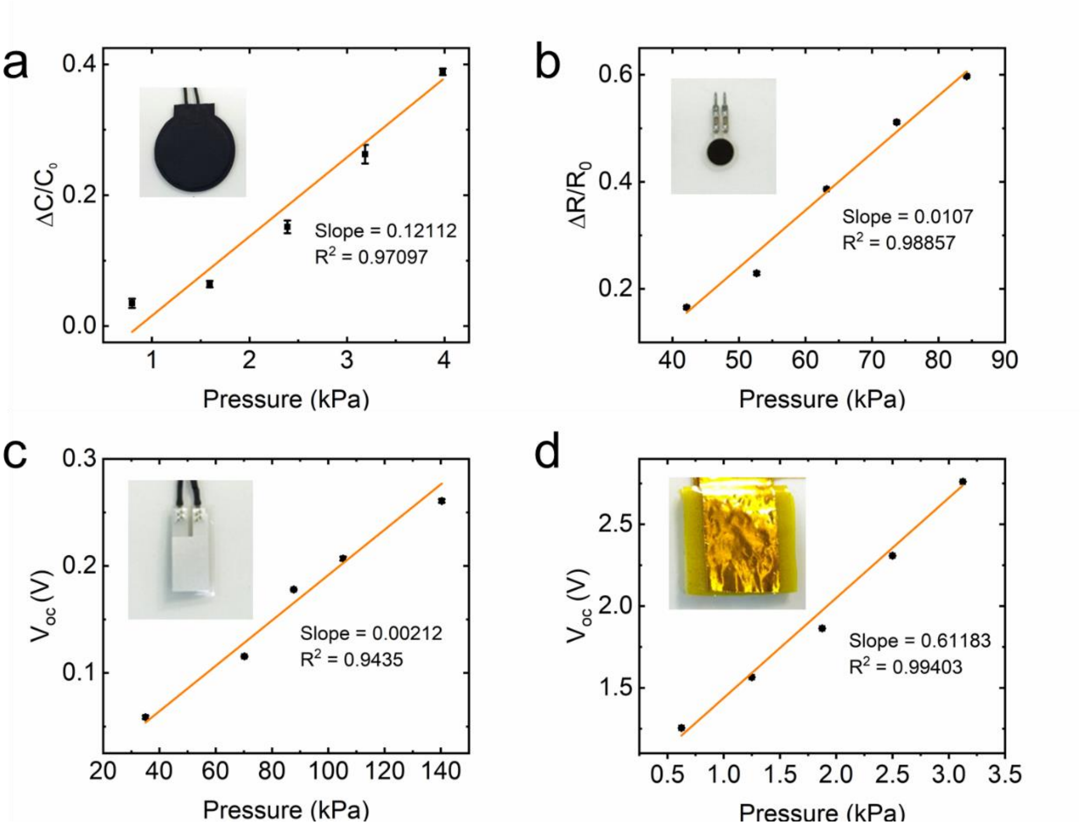

Supplementary Fig. 4 Sensitivities of sensors. a. sensitivity of capacitive sensor. b. sensitivity of piezoresistive sensor. c. sensitivity of piezoelectric sensor. d. sensitivity of triboelectric sensor.

Model	Sensitivity	Trigger point	Cost	Type
ESPB-01	0.12112 kPa^{-1}	1 N	￥288/ ＄45.1	capacitive
DF9-16	0.0107 kPa^{-1}	4 N	￥13/ ＄2.0	piezoresistive
LDT0-028K	0.00212 V/kPa	10 N	￥12/ ＄1.9	piezoelectric
This work	0.61183 V/kPa / 0.514 kPa^{-1}	1 N	￥2/ ＄0.31	triboelectric

Supplementary Tab. 2 Sensitivity, trigger point, cost of different sensors.

Supplementary note 4: The normalized waveform of main vowels

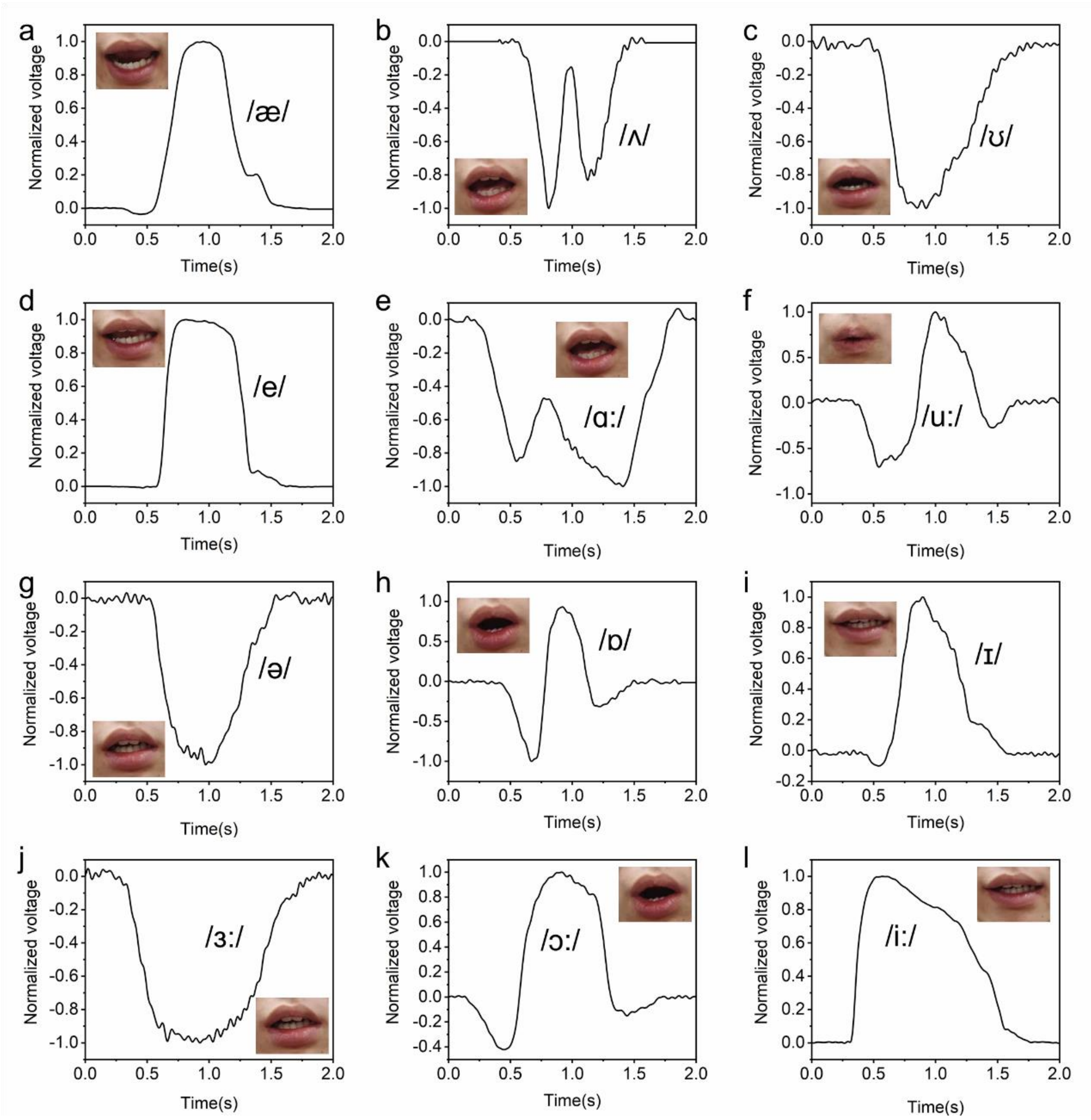

Supplementary Fig. 5 The normalized lip-motion voltage waveforms of 12 vowels (a-l) and related mouth shapes

Supplementary note 5: the characteristics of lip motion signals

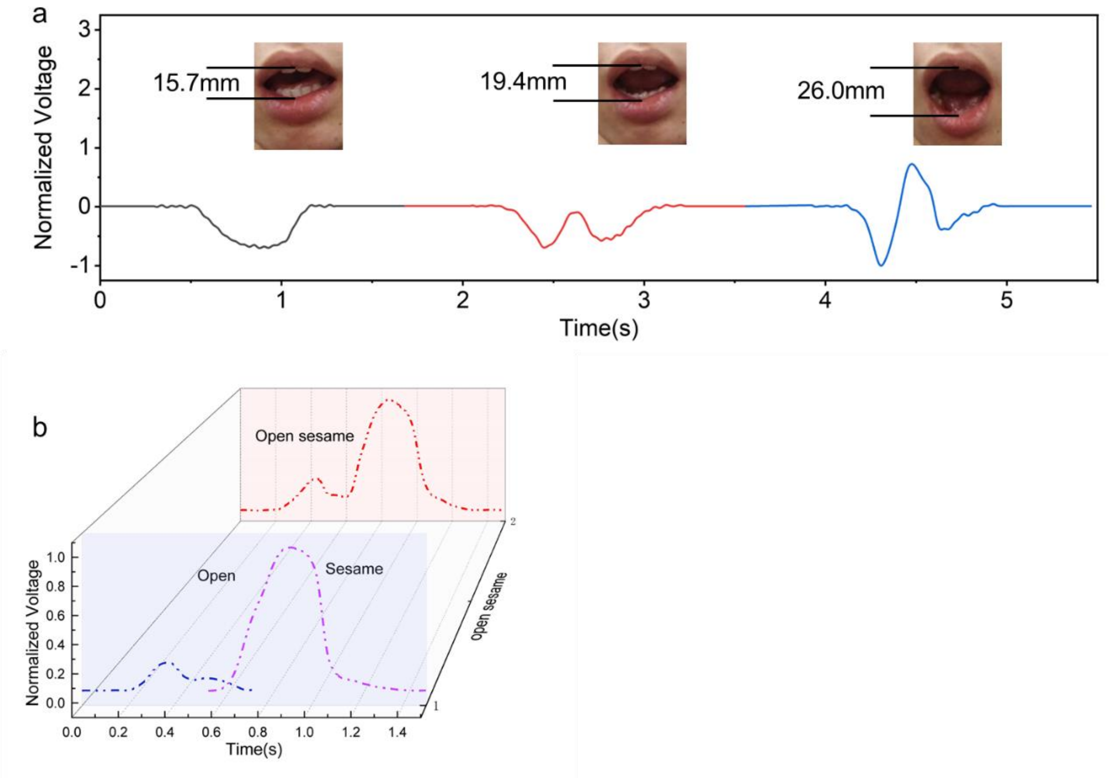

Supplementary Fig. 6 The characteristics of lip motion signals (a) Signals corresponding to the same pronunciation in different mouth opening size. (b) The combination and decomposed lip-motion signals of “Open”, “Sesame”, and “Open sesame”.

Supplementary note 6: Voltage data manipulation

Taking the normalized curves in Fig.6(b) for example, the obtained signals are filtered with a cut-off frequency of 20 Hz to filter out the power-frequency electromagnetic interference; the obtained signals are intercepted and the baseline is subtracted to reduce the baseline drift caused by ultra-low frequency noises. The baseline is determined by connecting the first point of the intercepted signal to the last point. In order to reduce the difference of voltage amplitude in different lip-motion recording processes, the obtained signals were normalized, and the absolute value of each point was normalized into the interval [0,1].

Supplementary note 7: setup of the neural network classifier.

In our method, for each class, the model learns a prototype in the deep feature space. In the classification stage, classification is performed by calculating the Euclidean distance between the feature representation of the sample and the class prototype. Specifically, the feature mapping

function is defined as: $f_\theta(x): \mathbb{R}^D \rightarrow \mathbb{R}^d$, where is the parameters of the feature extractor, D is the dimension of input space, and d is the dimension of the deep feature space. The corresponding prototype for each category is $\mu_i,\ i \in (1,\ldots C)$, in which C is the number of training classes. The probability of sample *(x,y)* belonging to class i is:

$$p(x \in \mu_i|x) = \frac{exp(-d(f(x),\mu_i))}{\sum_{c=1}^{C} exp(-d(f(x),\mu_c)} \quad (1)$$

Among them

$$d(f(x),\mu_i) = \|f(x) - \mu_i\|_2^2 \quad (2)$$

is the distance in feature space between the sample *(x,y)* and the prototype of class i. Based on the probability, the cross-entropy loss is:

$$l((x,y);\theta,\mu) = -log(p(y\,|\,x)) = -log(p(x \in \mu_i|x)) = -log\left(\frac{exp(-d(f(x),\mu_i))}{\sum_{c=1}^{C} exp(-d(f(x),\mu_c)}\right) \quad (3)$$

In addition, the following regularization term is added to learn compact feature representations for each class:

$$pl\big((x,y);\theta,\mu\big) = d(f(x),\mu_i) = \|f(x) - \mu_i\|_2^2 \quad (4)$$

The final learning objective of our method is as follows: $\mathrm{loss}((x,y);\theta,\mu) = l((x,y);\theta,\mu) + \alpha \cdot pl((x,y);\theta,\mu)$, where α is a hyper-parameter balancing the trade-off of each term.

Implementation details: We train all the models with batch size 50 and Adam optimizer[13] with 0.001 initial learning rate.

Supplementary note 8: Data collection process

Take the word "apple" for example. The participant speaks "apple" 150 times with a sampling rate of 500 Hz. The participant speaks the word for 15 times as a group and 10 groups in total; the participant can have a rest between groups. To control the rhythm of speech, a counter (15 counts at 4s intervals) is used to remind the participant. Then the signals are preprocessed for data recognition.

Supplementary note 9: Data preprocessing for machine learning

The data preprocessing for machine learning mainly consist of two steps.

First, machine learning algorithms do not perform well when the features of input samples have very different scales. Therefore, the first step of data preprocessing is standardization, which is a commonly used strategy of feature scaling. Specifically, standardization subtracts the mean value, and divides by the variance to make the features distribution have unit variance. With standardization, the machine learning process could be much less affected by outliers.

Second, to train and evaluate the model, the collected data is divided into two sets: the training set and the testing set. The total samples (2000 samples) are shuffled firstly. The first 1600 samples are viewed as the training set, and the remaining samples are viewed as the testing set.

After data preprocessing, the training set is processed by the machine learning algorithm.

Supplementary note 10: the chosen words and phrases

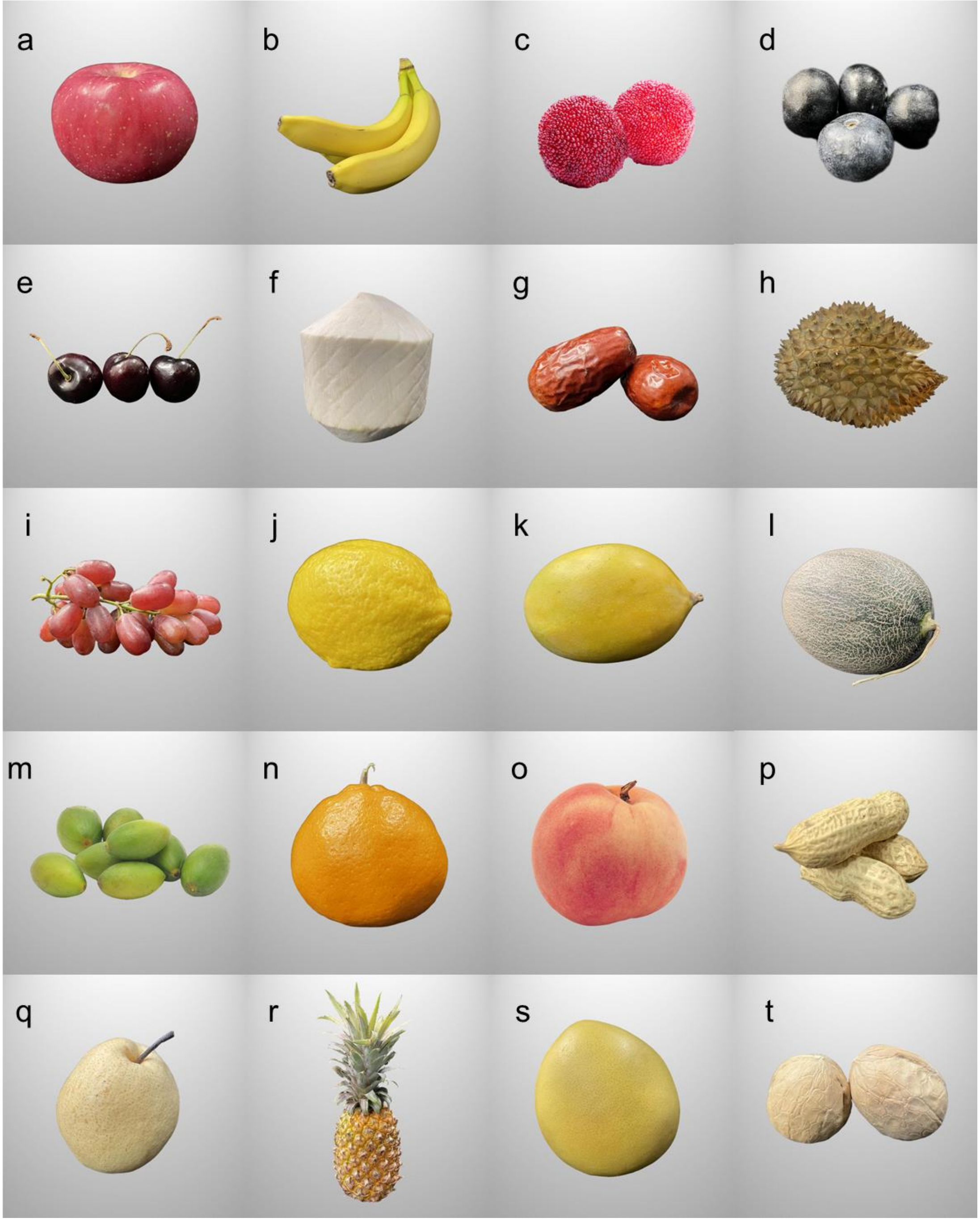

Supplementary Fig. 7 Words list (20 fruit photographs, a-t) selected for collection and training.

Apple	Banana	Bennet	Berry
/ˈæpl/	/bəˈnɑːnə/	/'benɪt/	/ˈberi/

**Cherry**	**Coconut**	**Date**	**Durian**
/ˈtʃeri/	/ˈkəʊkənʌt/	/deɪt/	/ˈdʊəriən/
**Grape**	**Lemon**	**Mango**	**Melon**
/greɪp/	/ˈlemən/	/ˈmæŋgəʊ/	/ˈmelən/
**Olive**	**Orange**	**Peach**	**Peanut**
/ˈɒlɪv/	/ˈɒrɪndʒ/	/piːtʃ/	/ˈpiːnʌt/
**Pear**	**Pineapple**	**Pomelo**	**Walnut**
/peə(r)/	/ˈpaɪnæpl/	/ˈpɒmələʊ/	/ˈwɔːlnʌt/

Supplementary Tab. 3 The chosen words and phonetic symbols

**1**	**Nice**	**To**	**Meet**	**You**
	/naɪs/	/tə; tu; tuː/	/miːt/	/ju; juː/
**2**	**Open**	**Sesame**		
	/ˈəʊpən/	/ˈsesəmi/		
**3**	**Zhi 芝**	**Ma 麻**	**Kai 开**	**Men 门**
	/ʈʂʅ/	/ma/	/kʰaɪ/	/mən/

Supplementary Tab. 4 The chosen phrases and phonetic symbols of each word. English uses British phonetic alphabet, and Chinese pinyin uses International Pinyin (IPA). ‘Zhima kaimen’ is the ‘Open saseme’ in Chinese.

Supplementary note 11：Waveform of the 20 fruit words

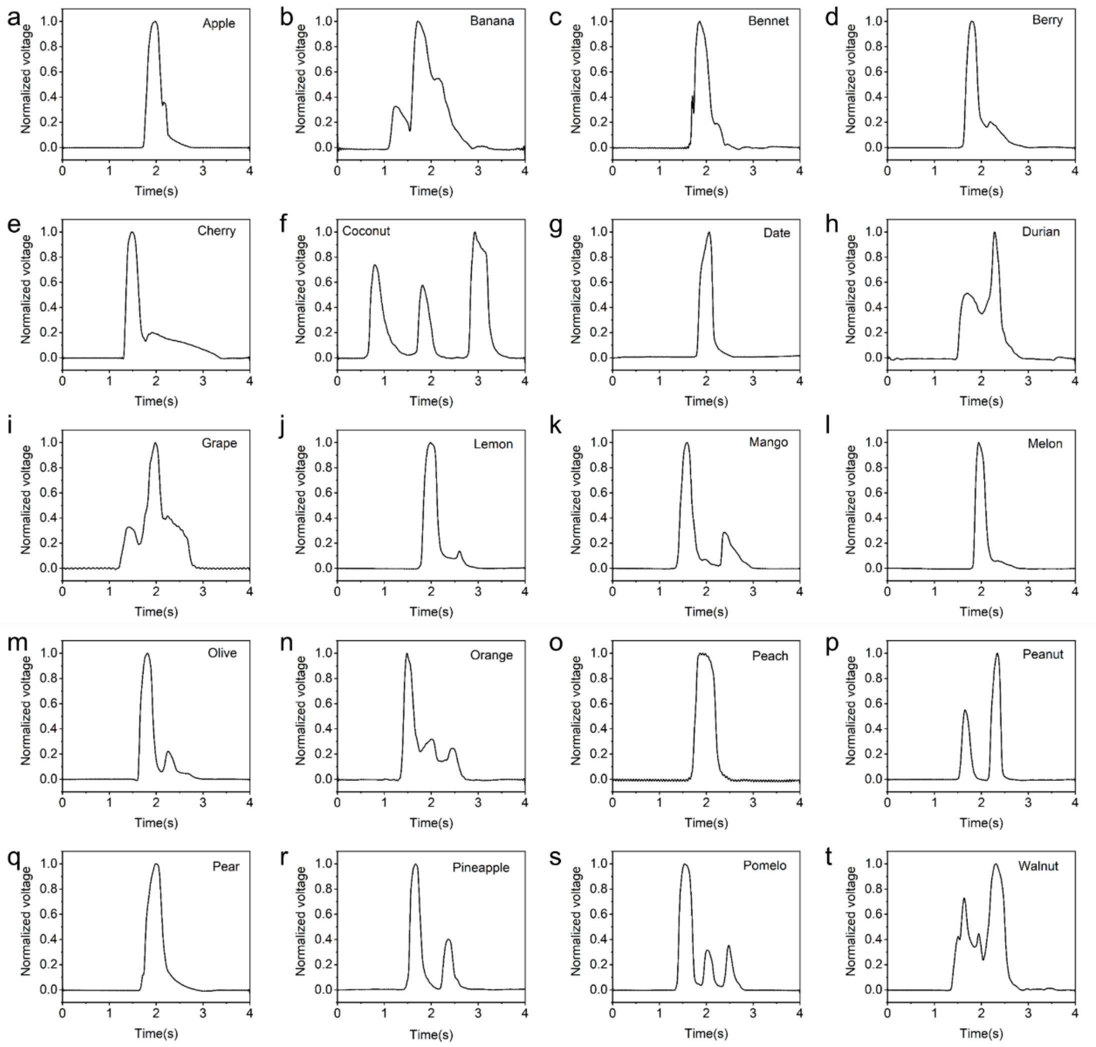

Supplementary Fig. 8 The normalized lip-motion voltage waveforms of 20 words (a-t).

Supplementary note 12: impact of hyper-parameters of dilated RNN

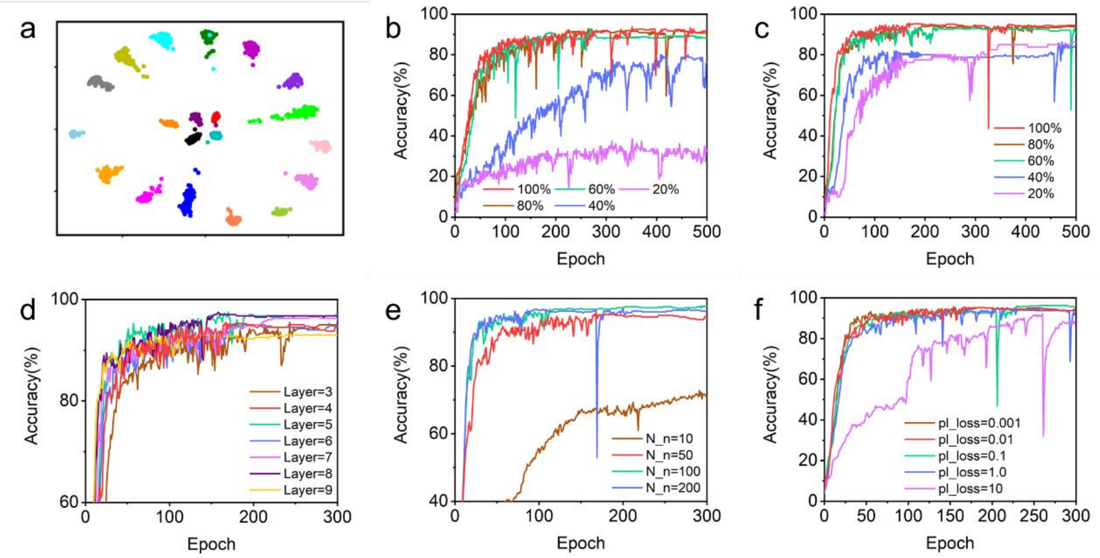

Supplementary Fig. 9 The impact of hyper-parameters including the number of layers and neurons in each layer of the test. (a) Visualization of the two-dimensional features of the dilated recurrent neural network based on softmax classifier. (b) The accuracy curve of the softmax-based model when the training sample is gradually reduced. (c) The accuracy curve of the prototype-based model when the training sample is gradually reduced. (d)The accuracy curve based on prototype learning with different number of layers in the dilated RNN. (e) The accuracy curve based on prototype learning under different numbers of hidden layer neurons. (f) The accuracy curve based on prototype learning under different prototype regularization coefficients.

Supplementary note 13: training results of the dilated RNN

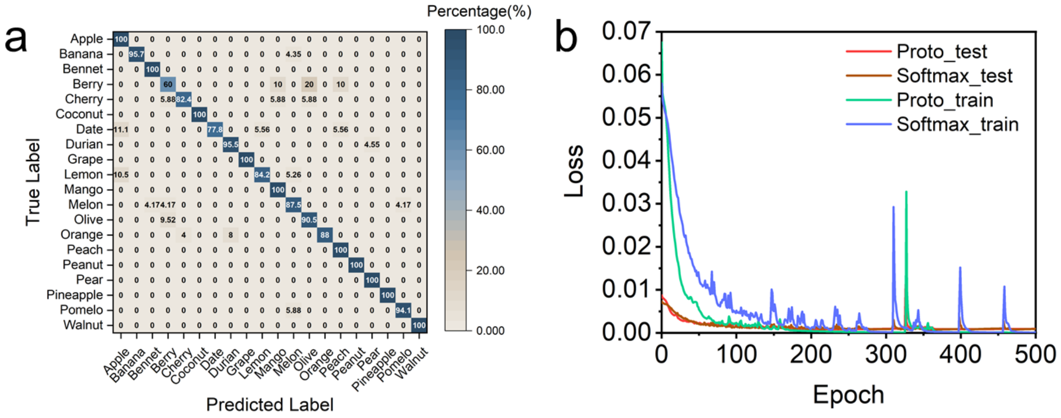

Supplementary Fig. 10 Training results of the dilated RNN. (a)The confusion matrix for lip-motion signals of 20 classes with softmax model. (b) The comparison of loss in prototype and softmax training and testing.

Supplementary note 14: analysis of the lip-motion signals.

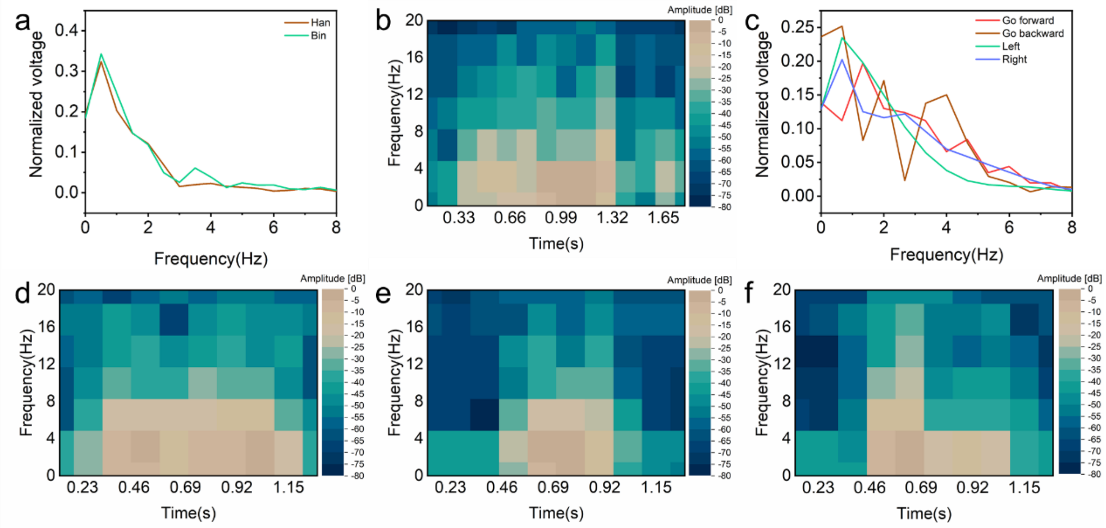

Supplementary Fig. 11 The analysis of the lip-motion signals. (a)The comparison of lip-motion signals from participants (Han and Bin) in frequency domain. (b) STFT analysis of the lip motion signals from Bin. (c) The comparison of lip-motion signals from Han in frequency domain. (d-f) STFT analysis of the lip motion signals of 'Go backward', 'Left' and 'Right' from Han.

Supplementary References

1. Ahmed A*, et al.* Triboelectric Nanogenerator versus Piezoelectric Generator at Low Frequency (<4 Hz): A Quantitative Comparison. *iScience* **23**, 101286 (2020).
2. Wu X, Han Y, Zhang X, Zhou Z, Lu C. Large-Area Compliant, Low-Cost, and Versatile Pressure-Sensing Platform Based on Microcrack-Designed Carbon Black@Polyurethane Sponge for Human-Machine Interfacing. *Advanced Functional Materials* **26**, 6246-6256 (2016).
3. Lou C*, et al.* A Graphene-Based Flexible Pressure Sensor with Applications to Plantar Pressure Measurement and Gait Analysis. *Materials (Basel)* **10**, (2017).
4. Kim K, Jung M, Jeon S, Bae J. Robust and scalable three-dimensional spacer textile pressure sensor for human motion detection. *Smart Materials and Structures* **28**, (2019).
5. Liu H*, et al.* Ionogel infiltrated paper as flexible electrode for wearable all-paper based sensors in active and passive modes. *Nano Energy* **66**, (2019).
6. Park SW, Das PS, Chhetry A, Park JY. A Flexible Capacitive Pressure Sensor for Wearable Respiration Monitoring System. *IEEE Sensors Journal*, 1-1 (2017).
7. Sheng L, Teo S, Liu J. Liquid-Metal-Painted Stretchable Capacitor Sensors for Wearable Healthcare Electronics. *Journal of Medical and Biological Engineering* **36**, 265-272 (2016).
8. Joseph J, Singh S, Vanjari S. Leveraging Innate Piezoelectricity of Ultra-Smooth Silk Thin Films for Flexible and Wearable Sensor Applications. *IEEE Sens J* **17**, 8306–8313 (2017).
9. Ghosh SK, Mandal D. Sustainable Energy Generation from Piezoelectric Biomaterial for

Noninvasive Physiological Signal Monitoring. *ACS Sustainable Chemistry & Engineering* **5**, 8836-8843 (2017).

10. Guo W*, et al.* Wireless piezoelectric devices based on electrospun PVDF/BaTiO3 NW nanocomposite fibers for human motion monitoring. *Nanoscale* **10**, 17751-17760 (2018).
11. Pu X*, et al.* Ultrastretchable, transparent triboelectric nanogenerator as electronic skin for biomechanical energy harvesting and tactile sensing. *Science Advances* **3**, e1700015 (2017).
12. Rasel MS*, et al.* An impedance tunable and highly efficient triboelectric nanogenerator for large-scale, ultra-sensitive pressure sensing applications. *Nano Energy* **49**, 603-613 (2018).
13. Kingma D, Ba J. Adam: A Method for Stochastic Optimization. *arXiv preprint arXiv:14126980*, (2014).
