# Supplementary material for: Decoding lip language using triboelectric sensors with deep learning
Source: Nat Commun. 2022 Mar 17;13:1401. doi: 10.1038/s41467-022-29083-0 (PMC8931018; doi:10.1038/s41467-022-29083-0)
Supplement: Supplementary file 2 — Description of Additional Supplementary Files [file 41467_2022_29083_MOESM2_ESM.pdf]

Description of Additional Supplementary Files

File Name: Supplementary Movie 1
Description: Lip-language decoding system (LLDS) used in personal identity verification

File Name: Supplementary Movie 2
Description: Lip-language decoding system (LLDS) used in direction control of toy car

File Name: Supplementary Movie 3
Description: Liplanguage decoding system (LLDS) used in lip-motion to voice
